# Tetraspanin 1 promotes endometriosis leading to ovarian clear cell carcinoma

**DOI:** 10.1002/1878-0261.12884

**Published:** 2021-01-07

**Authors:** Ha‐Yeon Shin, Wookyeom Yang, Doo Byung Chay, Eun‐ju Lee, Joon‐Yong Chung, Hyun‐Soo Kim, Jae‐Hoon Kim

**Affiliations:** ^1^ Department of Obstetrics and Gynecology Gangnam Severance Hospital Yonsei University College of Medicine Seoul Korea; ^2^ Department of Obstetrics and Gynecology Sahmyook Medical Center Seoul Korea; ^3^ Experimental Pathology Lab. Laboratory of Pathology National Cancer Institute National Institutes of Health Bethesda MD USA; ^4^ Department of Pathology and Translational Genomics Samsung Medical Center Sungkyunkwan University School of Medicine Seoul Korea

**Keywords:** AMP‐activated protein kinase, atypical endometriosis, endometriosis, ovarian clear cell carcinoma, tetraspanin 1

## Abstract

Ovarian clear cell carcinoma (OCCC) reportedly develops from endometriosis. However, the molecular mechanism underlying its malignant progression to OCCC remains elusive. This study aimed to identify an essential gene in the malignant transformation of endometriosis to OCCC. We performed RNA sequencing in formalin‐fixed, paraffin‐embedded (FFPE) tissues of endometriosis (*n* = 9), atypical endometriosis (AtyEm) (*n* = 18), adjacent endometriosis to OCCC (AdjEm) (*n* = 7), and OCCC (*n* = 17). We found that tetraspanin 1 (*TSPAN1*) mRNA level was significantly increased by 2.4‐ (DESeq2) and 3.4‐fold (edgeR) in AtyEm and by 80.7‐ (DESeq2) and 101‐fold (edgeR) in OCCC relative to endometriosis. We confirmed that TSPAN1 protein level was similarly overexpressed in OCCC tissues and cell lines. In immortalized endometriosis cell lines, TSPAN1 overexpression enhanced cell growth and invasion. Mechanistically, TSPAN1 triggered AMP‐activated protein kinase (AMPK) activity, promoting endometriosis and cell growth. Upregulated levels of TSPAN1 are considered an early event in the development of high‐risk endometriosis that could progress to ovarian cancer. Our study suggests the potential of TSPAN1 as a screening candidate for high‐risk endometriosis.

## Graphical abstract

Endometriosis can progress toward ovarian clear cell carcinoma (OCCC). While investigating genes involved in this process, we confirmed that TSPAN1 expression increases during progression from endometriosis to OCCC. TSPAN1 promoted endometriotic cell growth through AMPK activity. These results indicate altered TSPAN1 expression levels as an early marker of malignant transformation that may enable screening for high‐risk endometriosis.

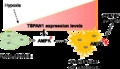

**Abbreviations**
AdjEm: adjacent endometriosis to OCCC
AMPK: AMP‐activated protein kinase
AtyEm: atypical endometriosis
DEGs: differentially expressed genes
EAOC: endometriosis‐associated ovarian cancers
FAP: fibroblast antigen protein
FFPE: formalin‐fixed paraffin‐embedded
GEO: Gene Expression Omnibus
OCCC: ovarian clear cell carcinoma
OEC: ovarian endometrioid carcinoma
RT‐PCR: reverse transcription PCR
TMA: tissue microarray
TSPAN1: tetraspanin 1

## 1 Introduction

Endometriosis, a benign gynecological disease, occurs in 10% of women of reproductive age. In endometriosis, endometrial cells exist outside of the endometrium of the uterus, resulting in chronic pain and infertility [1]. Although its cause is unknown, endometriosis is considered to be related to genetics, environment, immunology, angiogenesis, and endocrine disturbance [2]. Similar to malignant tumors, endometriosis also has characteristics of heterogeneity, adhesion, invasion, and metastasis in its benign form and can potentially transform into malignant cancer [2]. An association between endometriosis and ovarian cancer was initially proposed in 1925 [3]. Transition from endometriosis to ovarian cancer was confirmed in 1953 [4]. Meta‐analyses have reported that endometriosis is closely related to an increased risk of ovarian cancers when it occurs in its clear, endometrioid, and low‐grade serous form [5]. It is, therefore, important to screen for high‐risk endometriosis that could progress to cancer.

Ovarian clear cell carcinoma (OCCC) is a histological subtype of ovarian carcinoma showing distinctive epidemiological and clinical characteristics [6]. Among early‐stage ovarian cancer subtypes, OCCC has a favorable prognosis. However, among advanced‐stage ovarian cancer subtypes, OCCC has a poor prognosis due to resistance to antiplatinum and taxane‐based chemotherapy [7, 8]. OCCC has differing prevalence rates depending on race and ethnicity, and has greater prevalence in Asia than in western countries [9, 10]. A significant increase in the OCCC prevalence may be due to factors related to ovulation and menstruation [11]. OCCC is often diagnosed at an early‐stage, but it occurs in young women with an average age of 55 years than high‐grade serous ovarian carcinoma, which usually occurs at an average age of 64 years [9, 12].

Ovarian clear cell carcinoma is a common cancer that accounts for 40–50% of endometriosis‐associated ovarian cancers (EAOC) [13, 14, 15]. The histological precancerous lesion of OCCC is known as atypical endometriosis (AtyEm) [13] and is observed in 12–35% of ovarian endometriosis cases [2]. The transformation of endometriosis to malignant neoplasm involves an intermediate endometriosis lesion, such as AtyEm, and is further influenced by hormone levels, oxidative stress, genetic alteration, and immune dysregulation [16]. Activation of the oncogenic *KRAS* and *PI3K* pathways and inactivation of the tumor suppressor genes *PTEN* and *ARID1A* may be the main pathogenic mechanisms of this progression [17]. Accordingly, genetic mutations can explain their occurrence [18, 19]. In previous studies, gene expression profiling has been conducted to understand the molecular mechanisms associated with the transition from endometriosis to OCCC [20, 21]. However, these mechanisms have not yet been clearly explained.

In this study, we aimed to discover new mechanisms associated with the transformation of endometriosis to malignant OCCC. We sought to screen for high‐risk endometriosis and identify candidate genes that may serve as targets for preventive treatment, thus blocking the progression of endometriosis to cancerous tumors.

## 2 Materials and methods

### 2.1 Patients and tumor specimens

This study was approved by the Institutional Review Board of Gangnam Severance Hospital (3‐2015‐0298; Seoul, Republic of Korea). The experiments were undertaken with each patient's understanding and written consent, which was following the Declaration of Helsinki. All formalin‐fixed, paraffin‐embedded tissue samples were provided by the Korea Gynaecologic Cancer Bank through the Bio & Medical Technology Development Program of the Korean Ministry of Education, Science and Technology. For RNA sequencing, the FFPE tissue blocks comprised endometriosis (*n* = 9), AtyEm (*n* = 18), adjacent endometriosis to OCCC (AdjEm) (*n* = 7), OCCC (*n* = 17), and ovarian endometrioid carcinoma (OEC) (*n* = 12). For tissue microarray (TMA), the FFPE tissue blocks comprised endometriosis (*n* = 83), AtyEm (*n* = 13), AdjEm (*n* = 4), OCCC (*n* = 51), and OEC (*n* = 53).

### 2.2 Laser‐capture microdissection (LCM) and RNA extraction

All FFPE tissue slides were stained with hematoxylin and reviewed by an experienced gynecological pathologist. The selected lesions from whole tissues were excised by LCM. For LCM, the FFPE tissues were sectioned and placed on slides with polyethylene terephthalate membrane (Leica Microsystems Inc., Buffalo Grove, IL, USA). LCM was performed using a Leica AS LMD laser microdissection system (Leica Microsystems Inc.) according to the manufacturer’s instructions. A RNeasy FFPE kit (Qiagen, Valencia, CA, USA) was used to isolate total RNA from FFPE tissues according to the manufacturer’s instructions.

### 2.3 Ion AmpliSeq™ transcriptome library preparation

Total RNA was calculated as the percentage of RNA fragments longer than 200 nt using smear analysis of Agilent 2100 Bioanalyzer (Agilent Technologies, Santa Clara, CA, USA). DNA samples were quantified using a Qubit® dsDNA HS Assay Kit (Life Technologies, Carlsbad, CA, USA). An Ion AmpliSeqTM Transcriptome library was constructed with the Ion Transcriptome Human Gene Expression Kit (Life Technologies), as per the manufacturer’s protocol. Total RNA (10 ng) was reverse transcribed to synthesize cDNA by random priming. The cDNA product was used to amplify target genes using an Ion AmpliSeq™ Human Gene Expression Core Panel with an Ion AmpliSeq™ Library Kit Plus. After primer digestion, adapters and molecular barcodes were ligated to the amplicons, followed by magnetic bead purification. This library was amplified for five cycles and purified. Amplicon size and DNA concentration were measured using an Agilent High Sensitivity DNA Kit (Agilent Technologies) according to the manufacturer’s recommendation.

### 2.4 Ion proton sequencing

Sample emulsion PCR, emulsion breaking, and enrichment were performed using an Ion PITM Template OT2 200 Kit v3 (Life Technologies, Part #4488318 Rev. B.0) according to the manufacturer’s instructions. Multiple barcoded libraries were combined with equal molar ratios for one Ion PITM v2 chip. Two‐pooled Ion AmpliSeqTM Exome libraries were loaded onto a single Ion PITM v2 chip. Five‐pooled Ion AmpliSeq™ Transcriptome libraries were loaded onto a single Ion PITM v2 chip. Subsequent emulsion PCR and enrichment of the sequencing beads of the pooled libraries were performed using the Ion OneTouchTM system (Life Technologies) within approximately 7 h, according to the manufacturer’s protocol. Finally, 520 Flows sequencing was done on the Ion PITM v2 chip using Ion PITM Sequencing 200 Kit v3 (Life Technologies, Part #4488315 Rev. B.0) on the Ion ProtonTM sequencer (Life Technologies).

### 2.5 RNA sequencing read mapping and gene expression analysis

RNA sequencing reads were mapped to the human genome (hg19), and the read count for each gene was calculated. Each gene was normalized using read counts, and differentially expressed genes (DEGs) were analyzed using DESeq2 and edgeR. The complete datasets are available in the Gene Expression Omnibus database under accession number GSE157153. To obtain several candidate genes, we applied *P*‐values rather than adjusted *P*‐values. Because there was no or only one common gene when the fold change cut‐offs of > 2 and adjusted *P*‐values of < 0.05 or < 0.1 conditions were applied (Fig. S1). Consequently, we selected the 14 common genes according to the cutoff (2‐fold and *P*‐value < 0.05). A Venn diagram was drawn using a website (http://bioinformatics.psb.ugent.be/webtools/Venn/). A heat map was drawn as log values using cluster3.0 [22] and java treeview [23].

### 2.6 Cell culture

TOV‐21G, ES‐2, NIH3T3, and HS‐5 cell lines were purchased from the American Type Culture Collection (Manassas, VA). OVISE and OVTOKO cell lines were purchased from the Japanese Collection of Research Biosources Cell Bank (Osaka, Japan), the HEK293T cell line was purchased from System Biosciences (SBI Inc., Palo Alto, MA, USA), and the SNU‐251 cell line was purchased from the Korean Cell Line Bank (Seoul, Republic of Korea). TOV‐21G, OVISE, OVTOKO, and SNU‐251 cells were maintained in RPMI1640 supplemented with 1% penicillin/streptomycin. ES‐2 cells were maintained in McCoy’s 5A medium containing 10% FBS with 1% penicillin/streptomycin. 6045_SV40, 9585_SV40, HEK293T, NIH3T3, and HS‐5 cells were maintained in Dulbecco's modified Eagle's medium (DMEM) supplemented with 1% penicillin/streptomycin. 6595 and 6866_SV40 cells were maintained DMEM/F12 with 1% penicillin/streptomycin. All cell lines were cultured at 37 °C in a 5% CO_2_ atmosphere.

### 2.7 Plasmid construction and viral infection

For the generation of *TSPAN1* stable cell lines, cDNA encoding human *TSPAN1* was amplified using the primer set 5′‐AAGCTAGCATGCAGTGCTTCAGCTTC‐3′ (forward) and 5′‐TTGGATCCTTATTGTAGATTGCAGTA‐3′ (reverse). The amplified cDNA was cloned into NheI/BamHI restriction sites of the pCDH‐Promoter‐MCS‐EF1 Lentivector (System Biosciences, Mountain View, CA, USA), which has no green fluorescence protein (GFP) sequence. GFP deletion was conducted as follows: PCR products were amplified using the primer set 5′‐CCTACGCTAGACGCCACCATGACCGAGTACAAGCCC‐3′ (forward) and 5′‐GGGCTTGTACTCGGTCATGGTGGCGTCTAGCGTAGG‐3′ (reverse); pCDH‐promoter‐MCS‐EF1 Lentivector templates were selected by the restriction enzyme DpnI; and the empty Lentivector was used as a control for the stable cell lines. *TSPAN1*‐expressing stable cell lines were generated using the viral packaging plasmids composed of pCMV delta and pMDG. Virus particles were collected 48 h and 72 h post‐transfection. Positively infected cells were selected with 2 μg·mL^−1^ puromycin (Sigma‐Aldrich, St. Louis, MO, USA) for 15 days. pLenti CMV/TO SV40 small + Large T vector and human TERT were obtained from Addgene (Cambridge, MA, USA). HEK293T cells (1 × 10^6^) were co‐transfected with 2 μg lentiviral vector and 2 μg pPACKH1 Lentivector Packaging Kit (System Biosciences, Palo Alto). Crude virus supernatant (total collected viral medium of 10 mL) was collected 48 and 72 h after transfection. Cells were infected with 500 μL of the collected crude viral medium per dish, and the medium was changed with fresh ones after 24 h. During this process, the infected cells were not used with a selection marker as described previously [24].

### 2.8 siRNAs

*TSPAN1* (#1157352), *AMPK* (#5562‐1), *ARID1A* (#8289‐1, #8289‐2), and negative control (#SN‐1003) knockdown were conducted using predesigned siRNA sequences purchased from Bioneer (Daedeok‐gu, Daejeon, Republic of Korea). si*TSPAN1* transfection was performed using Lipofectamine RNAiMax (Thermo Scientific, Waltham, MA, USA), as per the manufacturer’s instructions. si*AMPK* transfection was carried out using G‐Fectin (Genolution Pharmaceuticals Inc., Seoul, Korea), as per the manufacturer’s instructions.

### 2.9 Immunohistochemistry

Paraffin tissue sections were deparaffinized in two changes of xylene, rehydrated in graded ethanol, and treated for 30 min with 3% H_2_O_2_ solution in methanol to block endogenous peroxidase activity. Then, the sections were incubated with mouse monoclonal anti‐human TSPAN1 antibody (Santa Cruz Biotechnology, Inc., Dallas, TX, USA; Cat# sc‐376551) for 1 h at RT, followed by detection using Dako LSAB+ (Dako, Glostrup, Denmark). The reaction product was developed with 3,3′‐diaminobenzidine chromogen solution (Dako). Sections were counterstained with hematoxylin and mounted in Faramount aqueous mounting medium (Dako). We used human small intestine tissue as positive controls for TSPAN1 staining. TSPAN1 staining was confirmed at the cytoplasmic and apical membrane of glandular cells (Fig. S2). TSPAN1 staining was scored as positive when tumor or epithelial cells showed cytoplasmic and membrane immunoreactivity. It was performed by a gynecological pathologist. TSPAN1 staining results were scored based on intensity (0 = negative, 1 = weak, 2 = moderate, 3 = strong) and the percentage of positive cells (0 = 0%, 1 = 1–25%, 2 = 26–50%, 3 = 51–100%), as described previously [25].

### 2.10 Real‐time and reverse transcription PCR

RNA extraction, cDNA synthesis, SYBR Green real‐time PCR, and quantification of mRNA were performed as described previously [26]. Reverse transcription PCR (RT‐PCR) was performed with Real‐taq polymerase (RBC Bioscience, New Taipei City, Taiwan) and a PCR machine (Eppendorf, Hamburg, Germany) according to the manufacturer's instructions. The PCR products were separated in 1% agarose gel at 30 V for 30 min and detected using a Gel Doc XR + imaging system (Bio‐Rad Laboratories, Inc, Hercules, CA, USA). The primers used real‐time PCR were as follows: for *TSPAN1*, forward 5′‐TGCCATGCAGTTTGTCAACG‐3′ and reverse 5′‐ACCATAGCAGCCCAGGAAAC‐3′; for *GAPDH*, forward 5′‐GAAGGTGAAGGTCGGAGTC‐3′ and reverse 5′‐GAAGATGGTGATGGGATTTC‐3′. The primers used RT‐PCR were as follows: *TSPAN1*, forward 5′‐AGCAAAAGGCTCACGACCAA‐3′ and reverse 5′‐CCCAATCACTGCTGCTTGCC‐3′; *hTERT*, forward 5′‐GAGAACAAGCTGTTTGCGGG‐3′; and *β*‐actin, forward 5′‐CTCGCCTTTGCCGATCC‐3′ and reverse 5′‐GGGGTACTTCAGGGTGAGGA‐3′. *hTERT*, forward 5’‐GAGAACAAGCTGTTTGCGGG‐3’ and reverse 5’‐AAGTTCACCACGCAGCCATA‐3’; *SV40 T*, forward 5’‐GCCCAGCCACTATAAGTACCA‐3’, *SV40 T* and reverse 5’‐CAAGCAACTCCAGCCATCCA‐3’; *β‐actin*, forward 5’‐CTCGCCTTTGCCGATCC‐3’ and reverse 5’‐GGGGTACTTCAGGGTGAGGA‐3’.

### 2.11 Protein extraction and immunoblotting

Western blot analysis was conducted as described previously [24]. Anti‐TSPAN1 (sc‐376551), anti‐α‐Actinin (sc‐17829), anti‐GAPDH (sc‐59541), anti‐Cytokeratin 7 (CK7; sc‐23879), anti‐Cytokeratin 18 (CK18; sc‐515852), anti‐Progesterone Receptor (PR; sc‐52), anti‐α‐Actinin (sc‐17829), and anti‐ARID1A (sc‐32761) antibodies were obtained from Santa Cruz Biotechnology, Inc, whereas anti‐pAMPK (Thr172, #2535), anti‐pACC (Ser79, #11818), anti‐ACC (#3662), anti‐pAKT (Ser473, #11818), anti‐ACC (#3662), anti‐pERK (Thr202/Tyr204 #4370), and anti‐ERK (#4695), anti‐Vimentin (#14472) and anti‐estrogen receptor alpha (ER‐alpha; #13258) antibodies were purchased from Cell Signaling Technology (Danvers, MA, USA). Anti‐AMPK alpha1 (#AF3197) was purchased from R&D Systems (Minneapolis, MN, USA). Anti‐estrogen receptor beta (ER‐beta; #PA1‐310B) was obtained from Thermo Scientific.

### 2.12 Gene Expression Omnibus (GEO) dataset analysis

Gene expression profiling data were obtained from the published microarray data of the GSE53012, GSE6008, GSE65986, and GSE29175 datasets from GEO. Identification of differentially expressed *TSPAN1* was conducted with a sorting tool based on microsoft excel software (Probe number: 209114_at in affymetrix human U133A platform; Probe number: ILMN_1747546 in Illumina HumanHT‐12 V3.0 expression beadchip; Gene accession number: NM_005727). Box plots and statistical analyses were performed using graphpad prism 5 software (GraphPad Software, Inc., La Jolla, CA, USA).

### 2.13 Immunofluorescence staining

6045_SV40 and 9585_SV40 cells were seeded in 24‐well cell culture plates until 30–40% confluence was reached. Cells were rinsed with PBS, fixed with ice‐cold methanol for 1 h at RT, and washed three times with PBS. The cells were incubated with TSPAN1 (1:100) with 1% bovine serum albumin in TBS containing 0.1% Tween‐20 (TBS‐T) solution overnight at 4 °C. After three TBS‐T washes, the cells were incubated with anti‐Mouse IgG conjugated to Alexa Fluor 488 dye (Cell Signaling) for 2 h at RT. Cells were washed three times with TBS‐T, and the nuclei were labeled with 1 g·mL^−1^ Hoechst 33342 (Sigma‐Aldrich) for 10 min at RT. Cells were washed three times with PBS for 5 min at RT. The cells were imaged using a microscope (Life Technologies, EVOS®FL Cell Imaging System).

### 2.14 WST‐1 assay

Cell proliferation was measured by WST assay (DaeilLab, Seoul, Republic of Korea). Briefly, cells were seeded at 0.1 × 10^4^ viable cells per well onto 96‐well plates at a final volume of 100 μL per well. Cells were incubated with WST‐1 at 37 °C for 2 h, and optical density (OD) values at 450 nm were recorded at days 0, 3, and 5 using a VERSA Max^TM^ (Bio‐Rad Laboratories, Inc.). For drug treatment, 6045_SV40 stable cells were seeded onto 96‐well plates at a density of 0.08 × 10^4^ viable cells per well in a final medium volume of 50 μL per well. After the cells were attached, they were treated dimethyl sulfoxide (DMSO), wortmannin (Sigma‐Aldrich), U0126 (Cell Signaling #9903), and Compound C (Selleckchem, Houston, TX, USA, S7306) with 50 μL medium. The medium with the drug was changed twice within 7 days. Cells were incubated with WST‐1 at 37 °C for 2 h, and OD values at 450 nm were recorded using VERSA MaxTM (Bio‐Rad Laboratories, Inc.).

### 2.15 Crystal violet staining

Cells were seeded at 0.5–2 × 10^4^ viable cells per well onto 24‐well plates. After the cells were attached, 20–50 nm si*TSPAN1*, si*AMPK alpha 1*, or siControl was transfected into them. Then, the cells were fixed using 10% acetic acid solution with 10% methanol at the indicated times, stained with 0.5% crystal violet for 1 h, photographed, and extracted using 1% SDS solution. Crystal violet extract from the cells was measured based on the absorbance at 595 nm with VERSA Max^TM^. All experiments were performed in triplicate.

### 2.16 Colony forming assay

For the long‐term colony forming assay, cells were seeded in 35‐mm dish at a density of 0.3–0.6 × 10^4^ viable cells per well, grown for 14 days, and fixed using 10% acetic acid solution with 10% methanol. For siRNA transfection, cells were seeded at 2 × 10^5^ viable cells per well onto 6‐well plates. Once cells were attached, 50 nm si*TSPAN1* and siControl were transfected into the cells. After 24 h, the cells were detached by trypsin‐EDTA (Hyclone), reseeded onto 35‐mm dish, and incubated for 10 days. Crystal violet staining was performed as mentioned above.

### 2.17 Cell invasion assay

A cell invasion assay was performed in an invasion chamber (Neuro Probe 48‐well Micro Chemotaxis Chamber, Neuro Probe, Inc., Gaithersburg, MD, USA) as described previously [27].

### 2.18 Endometriosis tissue and primary cell culture

Ectopic endometrium tissue samples were obtained from two patients who had histologically confirmed endometriosis. Endometrium tissue samples were obtained from two patients who did not have endometriosis. This study was approved by the Institutional Review Board of Gangnam Severance Hospital (3‐2019‐0320). The experiments were undertaken with each patient's understanding and written consent, which was following the Declaration of Helsinki. Biopsy materials were stored in sterile saline at 4 °C and transported to the laboratory. Fresh endometrial biopsy specimens were washed with PBS, chopped into small pieces, and centrifuged for 5 min at 1000 ***g***. After the supernatant was discarded, the tissues were incubated for 1 h at 37 °C in 1 mg·mL^−1^ of collagenase type IV. The dispersed cells were filtered through a 50‐μm cell strainer and were centrifuged for 5 min at 1000 ***g***. The collected cells were suspended in 1 mL DMEM/F12 or DMEM containing 10% FBS. Cells were cultured in an incubator at 37 °C in a 5% CO_2_ atmosphere. Images of the gap in the monolayers were captured (EVOS®FL Cell Imaging System).

### 2.19 STR profiling

Genomic DNA of the four cells was extracted using G‐spin^TM^ Total DNA Extraction Kit (iNtRON Biotechnology, Seoul, Republic of Korea). For STR profiling analysis, we assigned a cell line authentication service (Cosmo Genetech, Seoul, Republic of Korea). Genomic DNA was processed for STR profiling using a PowerPlex® 18D System (Promega, Madison, WI, USA), according to the manufacturer's instructions. After PCR amplification, the samples were analyzed using an ABI 3130xl Genetic Analyzer (Applied Biosystems, Foster City, CA, USA) and genemapper v5.0 software (Applied Biosystems). STR matching analysis was performed by Deutsche Sammlung von Mikroorganismen und Zellkulturen (DSMZ; www.dsmz.de).

### 2.20 Growth curve and doubling time

To count the number of cells, cells were seeded in a 6‐well plate at a density of 3 × 10^4^ cells per well, cultured for 8 days, and counted every 2 days. Subsequently, 10 μL of resuspended cells was measured using a LUNAII™ automated cell counter (Logos Biosystems, Inc., Anyang, Republic of Korea). All experiments were performed three times. The growth rate was estimated between 2 and 8 days using a previously described formula [24].

### 2.21 Immunocytochemistry

Cells were seeded in a Lab‐Tek Chamber slide (Nunc, Rochester, NY, USA) and fixed with ice‐cold methanol. Endogenous peroxidase activity was quenched by 3% hydrogen peroxide solution for 10 min, and the cells were washed three times in PBS. Nonspecific binding was prevented by incubation with 5% bovine serum albumin and 0.01% Triton X‐100 with TBS for 15 min. The sections were then incubated with a 1 : 20 dilution of the anti‐Fibroblast Marker antibody (Santa Cruz Biotechnology, sc‐73355) overnight at 4 °C. Antibody binding was detected using incubation with horseradish peroxidase‐conjugated secondary antibody at 37 °C for 1 h. Then, the sections were visualized by 3,3′‐diaminobenzidine solution (DakoKO, Seoul, Republic of Korea), counterstained lightly with hematoxylin (Sigma‐Aldrich), dehydrated with ethanol, and observed using inverted microscopy (Carl Zeiss Meditec AG, Suzhou, China).

### 2.22 Linear correlation by scatter plot

RNA sequencing reads were mapped to the human genome (hg19), and the read counts for each gene were calculated. Each gene was normalized using RPKM, and linear correlation was analyzed though scatter plots generated by the dnastar lasergene 15 software (DNASTAR, Madison, WI, USA).

### 2.23 Statistical analysis

Results are expressed as the means ± standard error (SE). Unpaired *t*‐tests were used to evaluate differences between two groups of variables. IHC scoring data was performed with Mann–Whitney test. Survival curve analysis was performed using the Kaplan–Meier method, and statistical significance was calculated using the log‐rank test. All analyses were carried out with graphpad prism 5 software. Differences were considered significant at *P < 0.05, **P < 0.01, ***P < 0.001, #*P* > 0.05.

## 3 Results

### 3.1 Gene expression profiling via DESeq2 and edgeR analysis

We obtained endometriosis and atypical endometriosis tissues from non‐OCCC patients. A total of 10 out of 17 OCCC patients had endometriosis around the cancer tissues, and we obtained endometriosis samples from 7 out of 10 OCCC with endometriosis lesions and used as AdjEm. AdjEm is histologically endometriosis; however, it is different from endometriosis and AtyEm in that it coexists with cancer tissues around it. Therefore, we included AdjEm in this study as it could explain the progression of OCCC. mRNAs were extracted only from the lesions excised from the FFPE tissues by LCM. The clinical information of the samples is listed in Table 1. The endometriosis group had the youngest mean age of 28 years old, while the other three groups had a mean age of 40. To identify the molecular changes responsible for the carcinogenic transformation of endometriosis to OCCC, RNA sequencing was performed. RNA sequencing results were compared between each group according to the linear correlation. Endometriosis and OCCC had the lowest linear correlation (*R* ^2^ = 0.8719), whereas AtyEm and AdjEm had the highest linear correlation (*R* ^2^ = 0.9746) (Fig. S3). These results indicate that AtyEm and AdjEm had the most similar gene profiles.

**Table 1:** Clinical characteristics of patients.

Characteristic	Endometriosis	AtyEm	AdjEm	OCCC	OEC
Number of cases					
*n* (%)	9 (17.64)	18 (35.29)	7 (13.72)	17 (33.33)	12 (19.05)
Age at presentation (years)					
Mean (SD)	27.78 (5.74)	40.00 (6.63)	44.57 (11.15)	43.59 (10.60)	48.50 (10.09)
FIGO stage, *n* (%)					
I/II	N/A	N/A	N/A	9 (41.17)	7 (58.33)
III/IV	N/A	N/A	N/A	2 (11.76)	1 (8.33)
Recurrent	N/A	N/A	N/A	4 (23.52)	1 (8.33)
Unknown	N/A	N/A	N/A	2 (11.76)	3 (25.00)
Tumor grade, *n* (%)					
Well + moderate	N/A	N/A	N/A	0 (00.00)	7 (58.33)
Poor	N/A	N/A	N/A	11 (64.70)	1 (8.33)
Unknown	N/A	N/A	N/A	6 (35.29)	4 (33.33)

N/A, not applicable; SD, Std. Deviation.

**Fig. 1:**
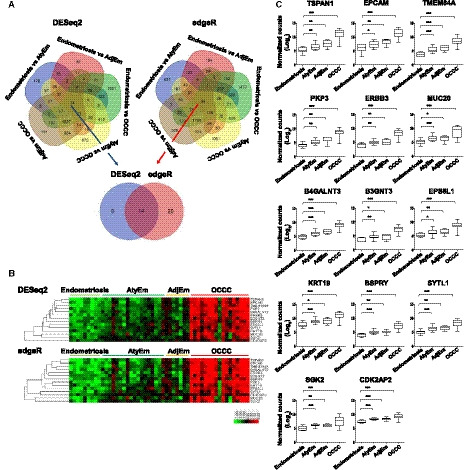
Identification of 14 upregulated genes by RNA sequencing. (A) Venn diagrams of the DEGs using DESeq2 and edgeR, based on five two‐group comparisons: Endometriosis versus AtyEm, Endometriosis versus AdjEm, Endometriosis versus OCCC, AtyEm versus OCCC, and AdjEm versus OCCC. DEGs in the common intersection are identified by DESeq2 (14 genes, blue arrow) and edgeR (34 genes, red arrow). Fourteen common genes were identified. (B) Heat map showing the expression of 14 genes in the four groups. (C) Individual gene expression profiles of the 14 genes in four groups. Normalized read counts were obtained using DESeq2. Unpaired *t*‐test was performed. *P < 0.05, **P < 0.01, ***P < 0.001.

To enhance the accuracy of DEG analysis, both DESeq2 and edgeR methods were used. DEGs were analyzed in five comparison groups: endometriosis versus AtyEm, endometriosis versus AdjEm, endometriosis versus OCCC, AtyEm versus OCCC, and AdjEm versus OCCC excluding AtyEm versus AdjEm. Next, to find the common DEGs in five comparison groups a Venn diagram was used; 14 and 34 genes were identified by DESeq2 and edgeR analysis, respectively. Then, 14 common genes were selected (i.e., *TSPAN1, EPCAM, TMEM84A, PKP3, ERBB3, MUC20, B4GALNT3, B3GNT3, EPS8L1, KRT19, BSPRY, SYTL1, SGK2,* and *CDK2P2*) (Fig. 1A). The heat map showed consistent increase in expression of all 14 genes from endometriosis to AtyEm and AdjEm and finally to OCCC (Fig. 1B). Through normalization counts (log2), the expression levels of 14 genes were higher in AtyEm and AdjEm than endometriosis, and in OCCC than AtyEm and AdjEm. All results were statistically significant (Fig. 1C). Fold changes and *P*‐values for all comparisons are listed in Table S1. Compared with endometriosis, *TSPAN1* (DESeq2; 80.7‐fold, edgeR; 101‐fold) had the most significant increase in OCCC among the 14 genes.

### 3.2 TSPAN1 expression is high in OCCC

To explore the function of TSPAN1, we cultured primary endometrial and endometriosis cells. hTERT and SV40 T antigens were expressed in these cells using lentivirus, resulting in immortalized cell lines (Fig. S4A) [28, 29, 30]. We identified the characteristics of four immortalized cell lines. SV40 expression was confirmed in cell lines 6866, 6045, and 9585, which used the SV40 T antigen (hereafter, 6866_SV40, 6045_SV40, and 9585_SV40). However, there was no detectable hTERT expression in the 6595 cell line using hTERT (Fig. S4B). Despite failure to induce immortalization, we used the 6595 cell line as a control for OCCC. The 6595 cell line grew the slowest than the cell lines immortalized with SV40, indicated by the doubling time (Fig. S4C). The expressions of the epithelial markers CK7 and CK18 were confirmed in 6045_SV40 and 9585_SV40 cells, whereas expression of the progesterone receptor B, estrogen receptor alpha and beta, and vimentin (Fig. S4D) were confirmed in all four cell lines. As the fibroblast antigen protein (FAP) was not detected by immunocytochemistry, fibroblasts were not mixed in all cell lines. NIH3T3 and HS‐5 were used as positive control cells for FAP (Fig. S4E). Lastly, DNA fingerprinting was performed in all four cell lines using the 18 short tandem repeat (STR) loci, and the STR profiles were compared using the Deutsche Sammlung von Mikroorganismen und Zellkulturen database, which confirmed that all four cell lines were novel (Table S2).

**Fig. 2:**
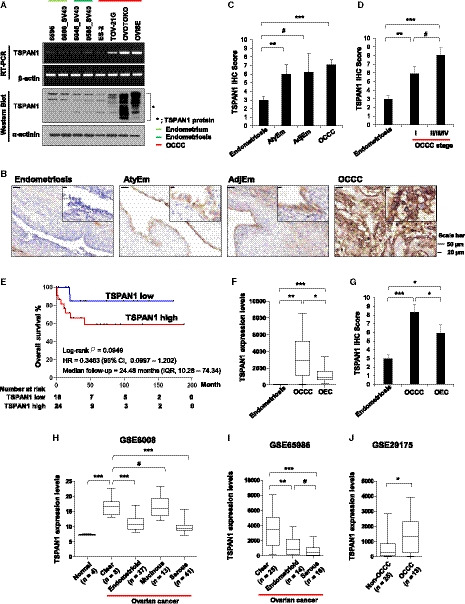
TSPAN1 is highly expressed in human OCCC cells and tissue specimens. (A) mRNA and protein levels of TSPAN1 were assessed using RT‐PCR (upper panel) and immunoblotting (lower panel) in cell lines. The expressions of β‐actin and α‐actinin were included as internal loading controls. (B) Representative immunohistochemical staining images of TSPAN1 in FFPE tissues. Scale bar = 50 or 10 μm; 200× or 1000× magnification. (C) Immunohistochemistry staining scores of TSPAN1 in Em, AtyEm, AdjEm, and OCCC. Data are expressed as the mean ± standard error (SE). Mann–Whitney test was performed. *P < 0.05, ***P < 0.001. (D) Immunohistochemistry staining scores of TSPAN1 according to OCCC stage. Data are expressed as the mean ± SE. Mann–Whitney test was performed. *P < 0.05, ***P < 0.001. (E) Overall survival curves for OCCC (*n* = 42) according to TSPAN1 expression using Kaplan–Meier curve analysis. (F, G) *TSPAN1* expression as shown by RNA sequencing analyzed by DESeq2 and the immunohistochemical scoring scores in endometriosis, OCCC, and OEC. *P < 0.05, **P < 0.01, ***P < 0.001. (H–J) mRNA expression levels of *TSPAN1* were analyzed in an ovarian cancer cell line (GSE29175) and ovarian cancer patient tissues (GSE6008 and GSE65986) in three GEO databases. Unpaired *t*‐test was performed. *P < 0.05, **P < 0.01, ***P < 0.001.

We investigated the TSPAN1 mRNA and protein expressions using our own endometrial cells (6595 and 6866_SV40), endometriosis cells (6045_SV40 and 9585_SV40), and OCCC cells (ES‐2, TOV‐21G, OVTOKO, and OVISE). TSPAN1 expression was higher in OCCC cells (TOV‐21G, OVTOKO, and OVISE, but not ES‐2) than in endometrial and endometriosis cells (Fig. 2A). Immunohistochemistry (Fig. 2B) confirmed that TSPAN1 protein expression was higher in AtyEm and OCCC than in endometriosis. However, there was no differential expression between endometriosis and AdjEm or among AtyEm, AdjEm, and OCCC cells (Fig. 2C). Additionally, TSPAN1 expression increased in stage I OCCC compared to that in endometriosis, but there was no difference between stages in OCCC (Fig. 2D). According to immunohistochemistry TSPAN1 analysis and patient clinical information, only the diagnostic category was statistically significant (*P* < 0.0001). The FIGO stage, chemoresponse, and CA‐125 had no statistical differences (Table S3). We also confirmed the tendency to have a poor prognosis in overall survival, but it was not statistically significant owing to the small sample size (Fig. 2E).

TSPAN1 has a different expression level in ovarian cancer subtypes. Mucinous and endometrioid subtypes of ovarian cancer tissues have higher TSPAN1 expressions than the serous subtype (*n* = 72) [31]. However, it has not been reported that results compared TSPAN1 expression levels in OCCC to other ovarian cancer subtypes. We additionally obtained RNA sequencing results of OEC. *TSPAN1* mRNA analysis revealed higher *TSPAN1* expression in clear cell type than in endometrioid types (*P* < 0.05) (Fig. 2F). Immunohistochemistry scoring revealed similar TSPAN1 protein expression (*P* < 0.05) (Fig. 2G). These results are supported by the three GEO dataset analysis (*P* < 0.05) (Fig. 2H–J).

### 3.3 TSPAN1 increase cell growth via AMPK phosphorylation in endometriosis cell lines

**Fig. 3:**
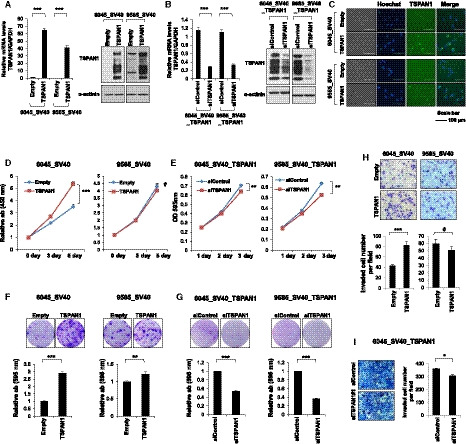
TSPAN1 increases cell proliferation in endometriosis cell lines. (A) TSPAN1 expression was detected by real‐time PCR (left panel) and immunoblotting (right panel) in stable cell lines, with α‐actinin as an internal loading control. Data are expressed as the mean ± standard error (SE); *n* = 3. Unpaired *t*‐test was performed. ***P < 0.001. (B) TSPAN1 expression was detected by real‐time PCR (left panel) and immunoblotting (right panel) after transient transfection of siControl or si*TSPAN1* in TSPAN1‐overexpressed stable cell lines, with α‐actinin as an internal loading control. Data are expressed as the mean ± SE; *n* = 3. Unpaired *t*‐test was performed. ***P < 0.001. (C) Immunofluorescent staining images of TSPAN1 (green) in stable cell lines. Nuclei are stained with Hoechst (blue). Scale bar = 100 μm, 400× magnification. Cell proliferation was determined via WST‐1 and (D) Crystal violet staining (E) at various times. Error bars represent mean ± SE; *n* = 3. Unpaired *t*‐test was performed. **P < 0.01, ***P < 0.001. (F, G) Colony forming assay was performed for the cell lines. Upper panel shows representative images; lower panel shows the relative absorbance at 595 nm. Error bars represent mean ± SE; *n* = 3. Unpaired *t*‐test was performed. **P < 0.01, ***P < 0.001. H–I. Cell invasion analysis of stable cell lines using a Matrigel invasion assay. Upper panel shows representative images (200× magnification); lower panel shows quantification of invasion experiments. Results are presented as the relative number of invading cells in five randomly selected fields. Error bars represent mean ± SE; *n* = 3. Unpaired *t*‐test was performed. *P < 0.05, ***P < 0.001.

To identify TSPAN1 function in endometriosis, TSPAN1‐overexpressing cell lines were produced using 6045_SV40 and 9585_SV40 cells. Each cell line was infected with TSPAN1‐overexpressing lentivirus and an empty lentivirus (control). It was confirmed that the overexpressing lines had higher TSPAN1 mRNA and protein levels than the control. The expression levels of the TSPAN1‐overexpressing cell lines decreased using siRNA (Fig. 3A,B). Immunofluorescence indicated TSPAN1 expression in the cytoplasm of the transfected 6045_SV40 and 9585_SV40 stable cell lines (Fig. 3C). To confirm that TSPAN1 affects the cell growth rate, the short‐term and long‐term effects on cell proliferation were examined in the 6045_SV40 and 9585_SV40 stable cell lines. TSPAN1‐overexpressing cells proliferated faster than the control in 6045_SV40 and 9585_SV40 stable cell lines. The 9585_SV40 stable cells showed faster growth only when tested for a long period of time (Fig. 3D,F). Next, *TSPAN1* knockdown reduced the growth rate of the TSPAN1‐overexpressing cell lines (Fig. 3E,G). Furthermore, Matrigel invasion assay identified an increase in invasion of 6045_SV40 stable cells in response to TSPAN1, whereas 9585_SV40 stable cells showed no change (Fig. 3H,I). These findings demonstrated that TSPAN1 increased endometriosis cell growth rate and affected cell invasion.

**Fig. 4:**
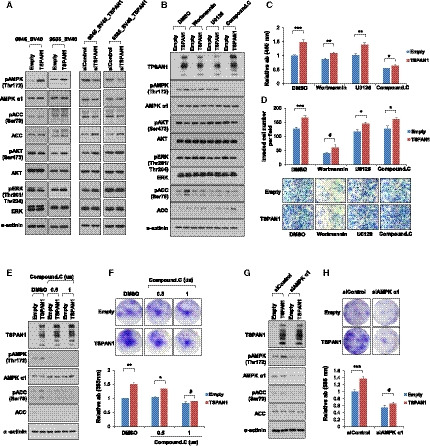
TSPAN1 increases cell growth through AMPK activity in the endometriosis cell line. (A) Protein levels were detected by immunoblotting in stable cell lines, with α‐actinin as an internal loading control (B) Protein levels were detected by immunoblotting after treatment of inhibitors in 6045_SV40 stable cell lines. (DMSO: control; 500 nm wortmannin: PI3 kinase inhibitor; 5 μm U0126: ERK inhibitor; 1 μm Compound C: AMPK inhibitor), with α‐actinin as an internal loading control. (C) Cell proliferation was determined via WST‐1 after treatment of inhibitors in 6045_SV40 stable cell lines. Unpaired *t*‐test was performed. *P < 0.05, **P < 0.01, ***P < 0.001. (D) Cell invasion analysis of stable cell lines using a Matrigel invasion assay after treating 6045_SV40 stable cells with inhibitors. Upper panel shows quantification of invasion experiments (200× magnification); lower panel shows representative images. Results are presented as the relative number of invading cells in five randomly selected fields. Error bars represent mean ± standard error (SE); *n* = 3. Unpaired *t*‐test was performed. *P < 0.05, ***P < 0.001. (E) Protein levels were confirmed by immunoblotting after treatment of DMSO and 0.5 and 1 μm of Compound C in 6045_SV40 stable cell lines, with α‐actinin as an internal loading control. (F) Cell proliferation was measured via crystal violet assay after treatment of DMSO and 0.5 and 1 μm of Compound C in 6045_SV40 stable cells. Upper panel shows representative images; lower panel shows the relative absorbance at 595 nm. Error bars represent mean ± SE; *n* = 3 Unpaired *t*‐test was performed. *P < 0.05, **P < 0.01. (G) Protein expressions were detected by immunoblotting after transient transfection of siControl or si*AMPK alpha1* in 6045_SV40 stable cells, with α‐actinin as an internal loading control. (H) Cell proliferation was measured via crystal violet assay after transient transfection of siControl or si*AMPK alpha1* in 6045_SV40 stable cells. Upper panel shows representative images; lower panel shows the relative absorbance at 595 nm. Data are expressed as the mean ± SE; *n* = 3. Unpaired *t*‐test was performed. ***P < 0.001.

To determine the mechanism of growth and invasion of endometriosis cells in response to TSPAN1, we measured the phosphorylation of major kinases associated with cell growth and survival. TSPAN1 overexpression in the 6045_SV40 and 9585_SV40 stable cell lines increased AMPK‐Thr172 phosphorylation, which was reduced upon si*TSPAN1* treatment. However, TSPAN1 expression did not induce a change in pAKT‐Ser473 and pERK‐Thr201/Thr204 (Fig. 4A). We treated the 6045_SV40 stable cell line with an AKT upstream kinase PI3K inhibitor (wortmannin), AMPK inhibitor (compound C), and ERK inhibitor (U0126). Wortmannin and Compound C treatments retarded the growth of the TSPAN1‐overexpressing cell lines, whereas U0126 treatment caused no change (Fig. 4C). Next, wortmannin greatly reduced cell invasion regardless of TSPAN1. When treated with U0126 and Compound C, TSPAN1‐overexpressing cell invasion did not decrease (Fig. 4D). Kinase activity was confirmed by their phosphorylation levels. AMPK and ERK activities were attenuated by their inhibitor, but AKT activity was maintained regardless of its inhibitor treatment (Fig. 4B). Wortmannin treatment did not reduce AKT‐Ser473 phosphorylation, but TSPAN1‐induced cell growth was attenuated. Therefore, it could be speculated that TSPAN1 is involved in cell growth through other PI3K pathways rather than PI3K/AKT signaling.

Notably, Compound C reduced AMPK‐Thr172 phosphorylation and TSPAN1‐induced cell growth. The crystal violet assay showed that the TSPAN1**‐**induced cell growth decreased depending on the Compound C concentration (Fig. 4E,F). Finally, the cell growth was assessed via the treatment of si*AMPK alpha1,* which attenuates AMPK‐Thr172 phosphorylation (Fig. 4G). The cell growth was accelerated in siControl cells in response to TSPAN1, but AMPK alpha1 knockdown cells showed no difference in growth (Fig. 4H). These results suggest that TSPAN1 induces cell growth via AMPK phosphorylation in endometriosis cells.

### 3.4 TSPAN1 knockdown reduce OCCC cell growth via a mechanism not involving AMPK

**Fig. 5:**
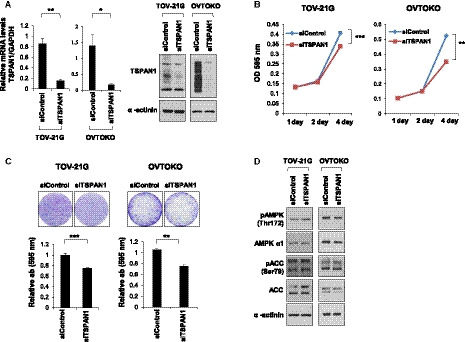
TSPAN1 knockdown decreases cell proliferation in OCCC cell lines. (A) TSPAN1 expression was detected by real‐time PCR (left panel) and immunoblotting (right panel) in TOV‐21G and OVTOKO cells, with α‐actinin as an internal loading control. Data are expressed as the mean ± standard error (SE); *n* = 3. Unpaired *t*‐test was performed. *P < 0.05, **P < 0.01. (B) Cell proliferation was determined via crystal violet staining at various times after transient transfection of siControl or si*TSPAN1* in TOV‐21G and OVTOKO cells. Error bars represent mean ± SE; *n* = 3. Unpaired *t*‐test was performed. **P < 0.01, ***P < 0.001. (C) Colony forming assay was performed in TOV‐21G and OVTOKO cells. Upper panel shows representative images; lower panel shows the relative absorbance at 595 nm. Error bars represent mean ± SE; *n* = 3. Unpaired *t*‐test was performed. **P < 0.01, ***P < 0.001. (D) Protein expressions were detected using immunoblotting after transient transfection of siControl or si*TSPAN1* in TOV‐21G and OVTOKO cells, with α‐actinin as an internal loading control.

To investigate whether TSPAN1 regulates the OCCC growth, si*TSPAN1* was transiently transfected into TOV‐21G and OVTOKO cells, followed by cell growth measurements. Similar to endometriosis cells, the growth rate of OCCC cell lines was delayed in response to reduced TSPAN1 expression (Fig. 5A–C). However, unlike in endometriosis cells, in TOV‐21G and OVTOKO cells, si*TSPAN1* did not cause a change in AMPK‐Thr172 phosphorylation compared with the siControl (Fig. 5D). Next, we established a TSPAN1‐overexpressing TOV‐21G cell line, which had lower TSPAN1 levels than OVTOKO (Fig. S5A). Consistent with the observation in si*TSPAN1*, while there was a difference in cell growth in the long‐term experiment, there was no effect on AMPK‐Thr172 phosphorylation when TSPAN1 was overexpressed (Fig. S5B–D). We speculate that when cells have been transformed into a malignant cell type such as OCCC, TSPAN1 regulates cell growth via mechanisms other than AMPK.

## 4 Discussion

Endometriosis is a common condition in young women of childbearing age [1]. In the surgical treatment of endometriosis, the ovaries are preserved because of their relationship with the secretion of female hormones [32]. However, as endometriosis may lead to the development of ovarian cancer [2], pre‐emptive therapy is necessary through high‐risk screening. Fortunately, because endometriosis tissue can be obtained by a simple surgical method, it is easy to apply pathological markers for endometriosis via tissue immunostaining. Thus, it is necessary to observe gene expression changes in endometriosis, AtyEm, AdjEm, and OCCC via RNA sequencing.

We used AtyEm and AdjEm as an intermediate stage between endometriosis and OCCC for RNA sequencing analysis. According to the unsupervised clustering results of the previously reported immune genes (*n* = 511), AtyEm (85%) shows a similar immune environment to cancer. This is because AtyEm cells were homogeneously clustered closer to EAOC than the endometrium [20]. OCCC‐associated endometriosis has abnormal gene expression that does not occur in endometriosis without cancer tissues [21]. We also showed highest linear correlation between AtyEm and AdjEm, and thus, the gene profiles of both groups were similar. Therefore, AtyEm and AdjEm exist in different environments and possess morphological differences. By extension, we suggest that these are intermediate stages representing gene changes, which may explain the carcinogenic process of OCCC. Fourteen common genes showing a pattern of a stepwise increase in expression from endometriosis to OCCC, while transitioning through AtyEm and AdjEm, can be considered as potential candidate gene markers for high‐risk endometriosis screening.

TMA staining showed that TSPAN1 has higher expression in AtyEm and early‐stage OCCC than in endometriosis (Fig. 2D). TSPAN1 therefore has potential as a pathological marker of high‐risk endometriosis. In addition, in this study, TSPAN1‐overexpressing endometriosis and OCCC cells had increased cell growth compared to control cells. Conversely, inhibition of cell growth was observed upon *TSPAN1* knockdown. TSPAN1, a member of the tetraspanin family, is highly expressed in many types of cancer, and increases cell growth, invasion, and migration in colon, cervical, pancreatic, and prostate cancers [33, 34, 35, 36]. TSPAN1 reportedly plays a role in increasing epithelial‐to‐mesenchymal transition and metastasis via the PI3K/AKT pathway in cholangiocarcinoma [37]. The PI3K/AKT pathway is activated in endometriosis [38, 39], and together with the ERK pathway, it enhances endometriotic stromal cell growth and survival [40]. However, in this study, TSPAN1‐overexpressing endometriosis cells did not have altered AKT and ERK activity, and their growth was promoted via AMPK activation. AMPK activity has the function of tumor suppression, which inhibits the anabolic process that maintains cancer cell proliferation. Conversely, AMPK has characteristics of a tumor promoter, allowing cancer cells to survive in an environment of metabolic stress or matrix detachment conditions. AMPK therefore exhibits a dual role in cancer survival [41, 42]. The results of the current study demonstrate that AMPK activity had a tumor‐promoting function and increased endometriosis cell growth in response to TSPAN1.

As endometrial cells leave the uterus, they are exposed to hypoxia. During this process, endometriosis is formed through complex survival processes such as steroidogenesis, angiogenesis, inflammation, and metabolic switches [43]. To withstand the hypoxic conditions, complex gene regulation is involved in endometriosis development [43]. Hypoxia is the main stimulus that activates AMPK [44, 45]. AMPK stimulates signaling essential for survival when cells are exposed to a variety of stressful environments, including hypoxia [46, 47]. Therefore, AMPK may be activated in endometriosis cells in various stressful environments, thus affecting cell survival and growth. We confirmed the *TSPAN1* expression change in hypoxic conditions through GSE53012 dataset [48] analysis. When prostate cancer PC‐3, ovarian cancer SKOV3, and melanoma WM793B cells were exposed to cycling (transient and intermittent) or chronic (prolonged) hypoxia, PC‐3 and SKOV3 cells showed higher *TSPAN1* expression levels (Fig. S6). Collectively, TSPAN1 is increased when endometriosis cells are exposed to stressful environments, thus affecting the endometriosis cell growth and invasion via AMPK activation.

*TSPAN1* knockdown in TOV‐21G and OVTOKO cells retarded cell growth. However, these cells exhibited no change in AMPK activation compared with endometriosis cells. Various gene mutations commonly found in OCCC may explain why AMPK was not activated by TSPAN1 in OCCC cells [17, 49]. The TOV‐21G, OVTOKO, and OVISE cells used in this study were OCCC cells with an *ARID1A* mutation (Fig. S7A) [50]. It was confirmed that AMPK activity (Thr172) was higher in OCCC cells than in endometriosis cells (Fig. S7A). To investigate whether AMPK activity is related to *ARID1A* mutation, we decreased the level of *ARID1A* protein using si*ARID1A* (Fig. S7B). When *ARID1A* was knocked down in endometriosis cells, AMPK‐Thr172 phosphorylation was elevated, as shown in western blot analysis (Fig. S7B). We thus confirmed that *ARID1A* mutation regulated AMPK activity. Additionally, TOV‐21G has mutations in *ARID1A*, *PIK3CA*, *PTEN*, and *KRAS*. Also, ES‐2 has mutations in *TP53* as well as *KRAS,* downstream of *BRAF*, although it possessed wild‐type *ARID1A* [51]. *KRAS* and *PTEN* mutations maintain cell growth by activating AMPK in astrocytic tumors [52]. Therefore, we expect that AMPK activity is maintained at high levels in OCCC cells due to various genetic mutations, AMPK activity does not further increase with an increase in TSPAN1.

Of 13 studies involving AMPK activity (Thr172) and overall, cancer‐specific, or progression‐free survival in various cancers, eight studies reported that AMPK activation led to improved prognosis [42]. However, studies of gastric cancer and prostate cancer have reported a link between pAMPK alpha1 and disease recurrence [42]. AMPK activation in breast cancer reduces disease‐specific and metastasis‐free survival [47]. Ovarian cancer has different clinical relationship with the expression of AMPK subunits according to histological subtype [26, 53]. Therefore, there is a need to compare AMPK activity and OCCC patient prognosis.

In summary, we have identified 14 genes that are importantly regulated during the transition phase of endometriosis to OCCC. Furthermore, we demonstrated that TSPAN1 is involved in endometriosis cell growth through AMPK activation. Unlike in endometriosis cells, the role of TSPAN1 is not carried out via AMPK activity in OCCC cells. However, considering that AMPK activity in OCCC is considerably high, it is predicted that if TSPAN1 and AMPK are simultaneously suppressed, this will inhibit OCCC growth. Therefore, this study has great significance in discovering the role of TSPAN1 in the malignant transformation of endometriosis into OCCC.

## 5 Conclusion

TSPAN1 was found to increase endometriosis cell growth and invasion by promoting AMPK activity. These results suggest that expression changes of TSPAN1 and other DEGs occurred early in the malignant transformation of endometriosis, showing the potential for screening for high‐risk endometriosis. Moreover, our findings enable the development of drugs that can aid in preventing the transformation of endometriosis into malignant cancer by controlling TSPAN1 levels and AMPK activity.

## Conflict of interest

The authors declare no conflict of interest.

## Author contributions

H‐YS performed the conception, study design, data analysis and interpretation, experimental work, and writing of the manuscript. WY performed the conception, study design, data interpretation, and technical support. DBC involved in the clinical information. E‐JL performed the technical support. J‐YC analyzed the data. H‐SK and J‐HK performed the study supervision, conception, review, and editing.

## Supporting information


**Fig. S1.** Venn diagram distribution of candidate genes based on adjusted p‐values.

**Fig. S2.** Immunohistochemical staining of TSPAN1 in human small intestine tissue.

**Fig. S3.** Linear correlation analysis using scatter plots of the six different two‐group comparisons.

**Fig. S4.** Characteristics of the immortalized endometrial (6595, 6866_SV40) and endometriosis (6045_SV40, 9585_SV40) cell lines.

**Fig. S5.** TSPAN1 increases cell growth but not cell invasion in the TOV‐21G stable cells.

**Fig. S6.** In GSE53012 analysis, *TSPAN1* expression was increased in cycling and chronic hypoxic conditions in PC‐3 and SKOV3, but not in WM793B.

**Fig. S7.** AMPK activity is high in OCCC and is increased by *ARID1A* knockdown.

**Table S1.** Tabular data shows the fold change of 14 genes in 5 different two‐group comparisons.
**Table S2.** STR profiling.
**Table S3.** TSPAN1 score of immunohistochemical staining.
