# Supplementary material for: Tetraspanin 1 promotes endometriosis leading to ovarian clear cell carcinoma
Source: Mol Oncol. 2021 Jan 7;15(4):987–1004. doi: 10.1002/1878-0261.12884 (PMC8024726; doi:10.1002/1878-0261.12884)
Supplement: Supplementary file 1 — Fig. S1. Venn diagram distribution of candidate genes based on adjusted p‐values. [file MOL2-15-987-s005.pdf]

A. The cutoff (> 2 fold change and adjusted p-value < 0.05)

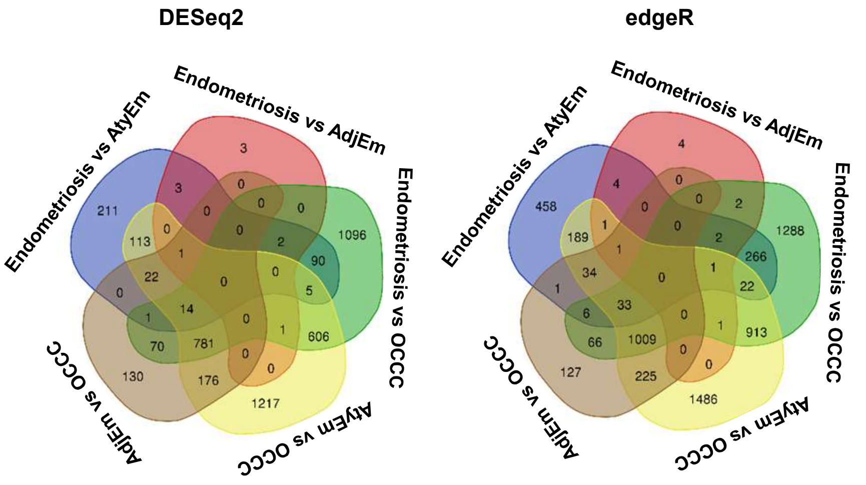

B. The cutoff (> 2 fold change and adjusted p-value < 0.1)

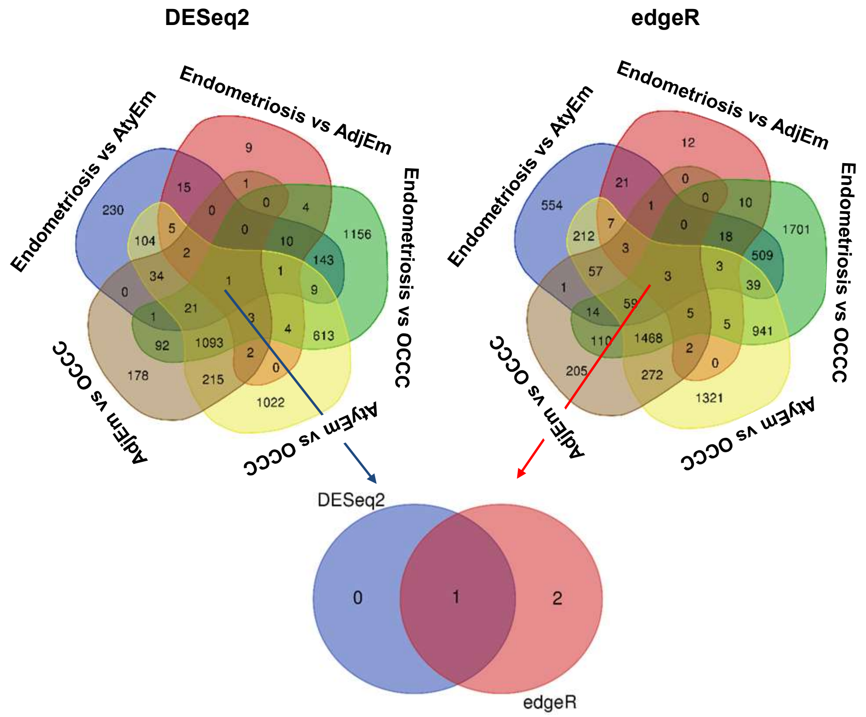

Fig. S1. Venn diagram distribution of candidate genes based on adjusted p-values.
