# Supplementary figures and images for: Tetraspanin 1 promotes endometriosis leading to ovarian clear cell carcinoma
Source: Mol Oncol. 2021 Jan 7;15(4):987–1004. doi: 10.1002/1878-0261.12884 (PMC8024726; doi:10.1002/1878-0261.12884)

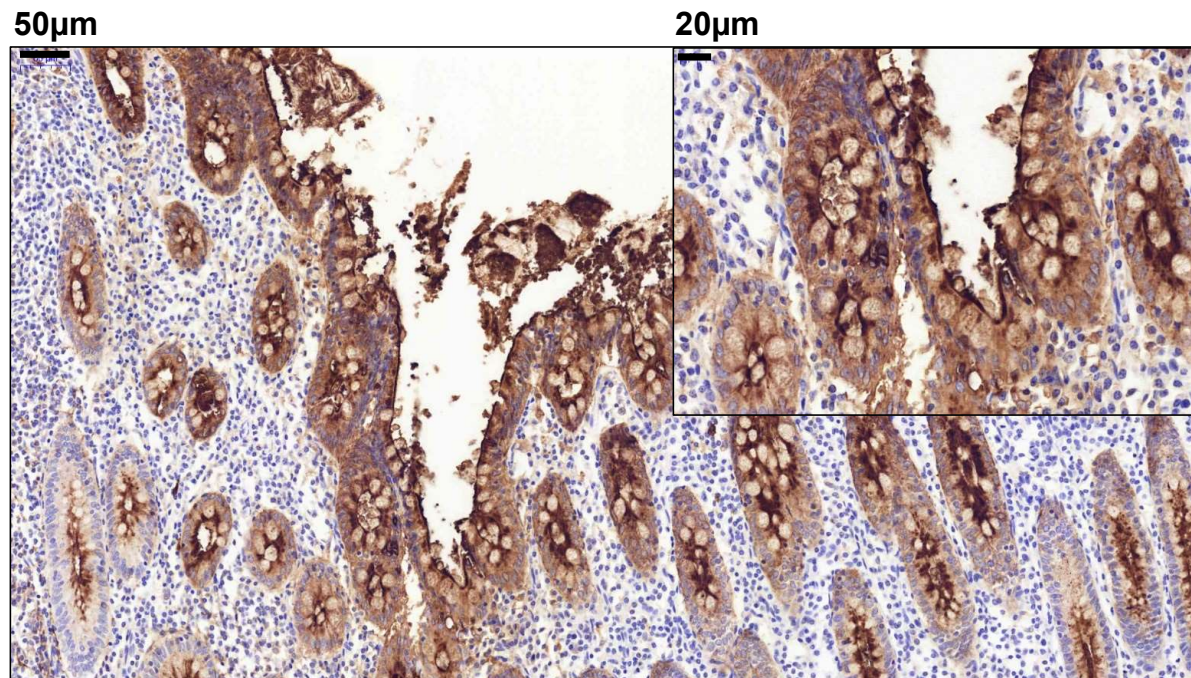

**Fig. S2. Immunohistochemical staining of TSPAN1 in human small intestine tissue.**

Supplement: Supplementary file 2 — Fig. S2. Immunohistochemical staining of TSPAN1 in human small intestine tissue. [file MOL2-15-987-s001.pdf]
