# Supplementary material for: Tetraspanin 1 promotes endometriosis leading to ovarian clear cell carcinoma
Source: Mol Oncol. 2021 Jan 7;15(4):987–1004. doi: 10.1002/1878-0261.12884 (PMC8024726; doi:10.1002/1878-0261.12884)
Supplement: Supplementary file 3 — Fig. S3. Linear correlation analysis using scatter plots of the six different two‐group comparisons. [file MOL2-15-987-s007.pdf]

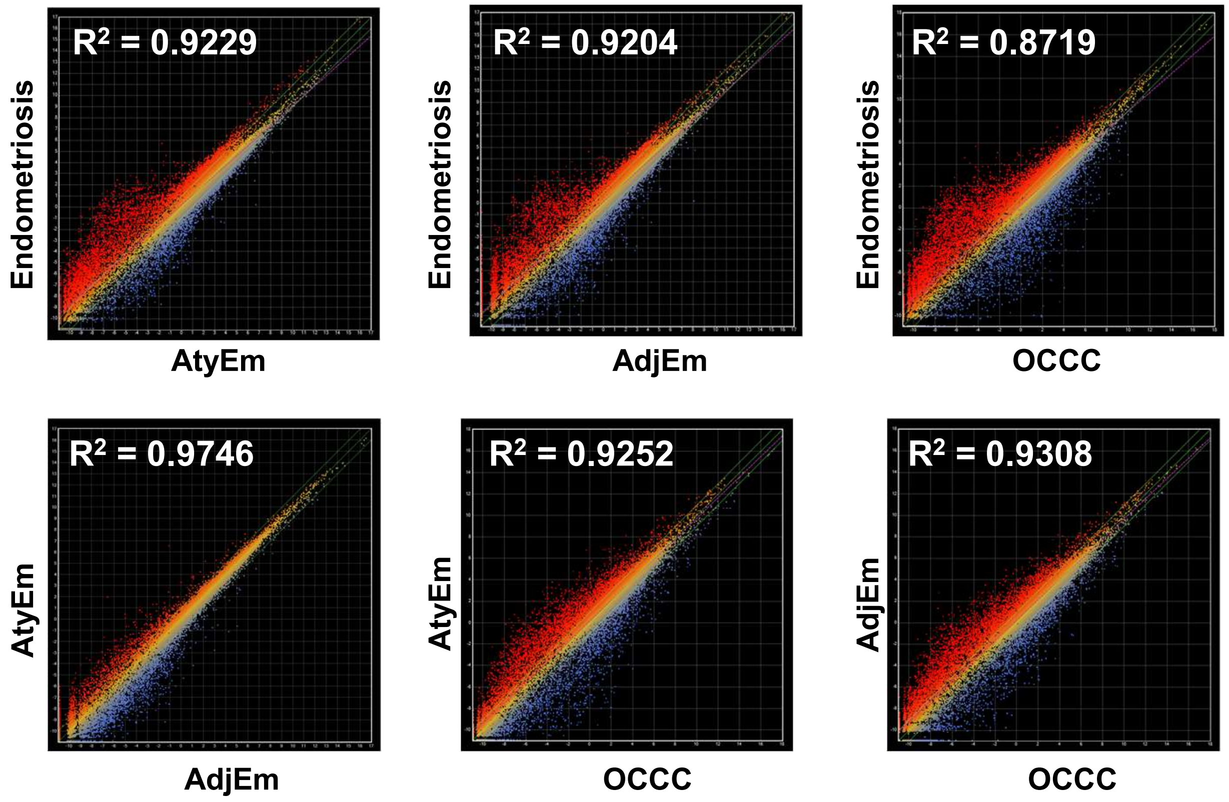

Fig. S3. Linear correlation analysis using scatter plots of the six different two-group comparisons.
