# Supplementary material for: Tetraspanin 1 promotes endometriosis leading to ovarian clear cell carcinoma
Source: Mol Oncol. 2021 Jan 7;15(4):987–1004. doi: 10.1002/1878-0261.12884 (PMC8024726; doi:10.1002/1878-0261.12884)
Supplement: Supplementary file 4 — Fig. S4. Characteristics of the immortalized endometrial (6595, 6866_SV40) and endometriosis (6045_SV40, 9585_SV40) cell lines. [file MOL2-15-987-s003.pdf]

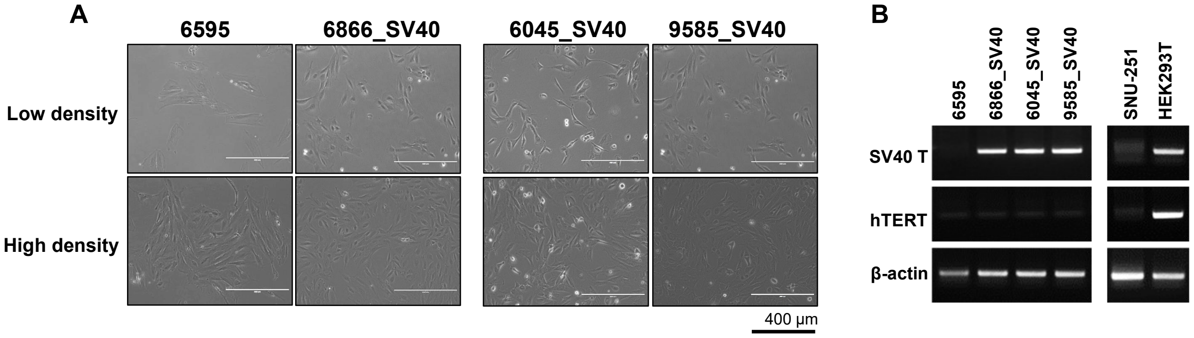

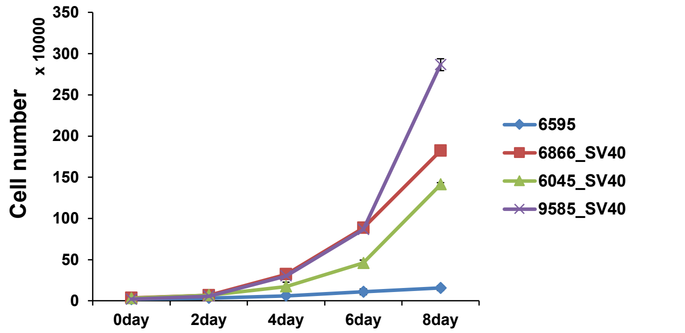

Cell line	6595	6866_SV40	6045_SV40	9585_SV40
Doubling Time (h)	65.38	33.42	35.12	27.33
S.D	13.73	4.30	2.81	2.81

S.D; Std.Deviation

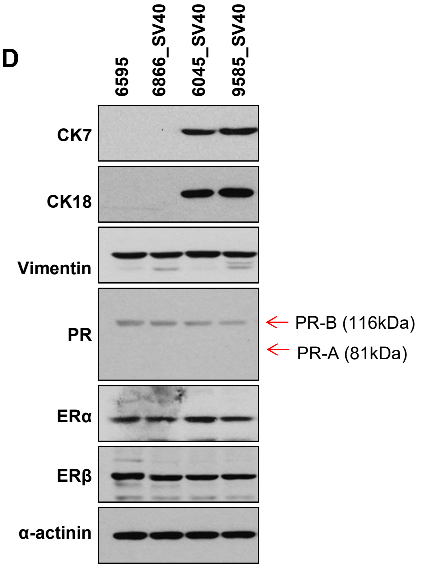

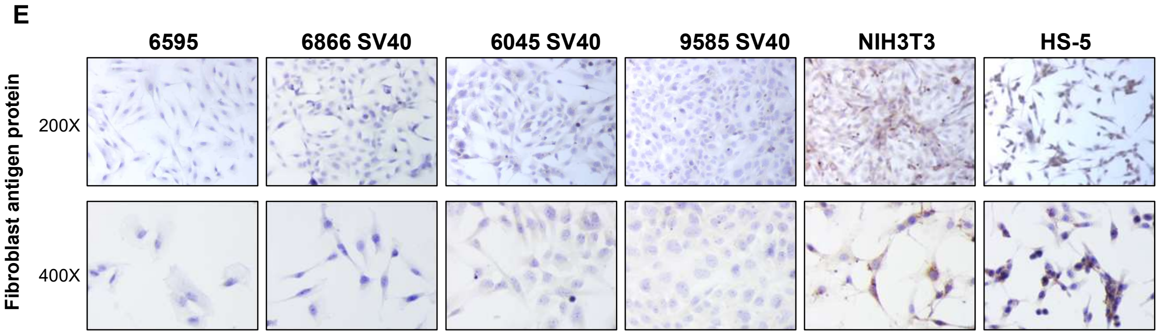

Fig. S4. Characteristics of the immortalized endometrial (6595, 6866_SV40) and endometriosis (6045_SV40, 9585_SV40) cell lines.

A. Representative images show the morphology of cells. Upper panel shows low density of cells; lower panel shows high density of cells. Scale bar: 400 μm, 100× magnification. **B.** mRNA levels of SV40 and hTERT were assessed using RT-PCR of cell lines. HEK293T and SNU-251 were the positive and negative controls, respectively, for SV40T and hTERT expression. β-actin expression was included as an internal loading control. **C.** Growth curves and doubling time of cells were determined by counting the cells every 2 d for 8 d. Error bars represent mean ± SD. Accompanying table lists the doubling time and SD. **D.** Protein expressions were detected using immunoblotting with α-actinin as an internal loading control. **E.** Representative immunocytochemical staining images of FAP in cell lines. NIH3T3 and HS-5 were the positive controls for FAP. 200× magnification, 400× magnification.
