# Supplementary material for: Tetraspanin 1 promotes endometriosis leading to ovarian clear cell carcinoma
Source: Mol Oncol. 2021 Jan 7;15(4):987–1004. doi: 10.1002/1878-0261.12884 (PMC8024726; doi:10.1002/1878-0261.12884)
Supplement: Supplementary file 5 — Fig. S5. TSPAN1 increases cell growth but not cell invasion in the TOV‐21G stable cells. [file MOL2-15-987-s004.pdf]

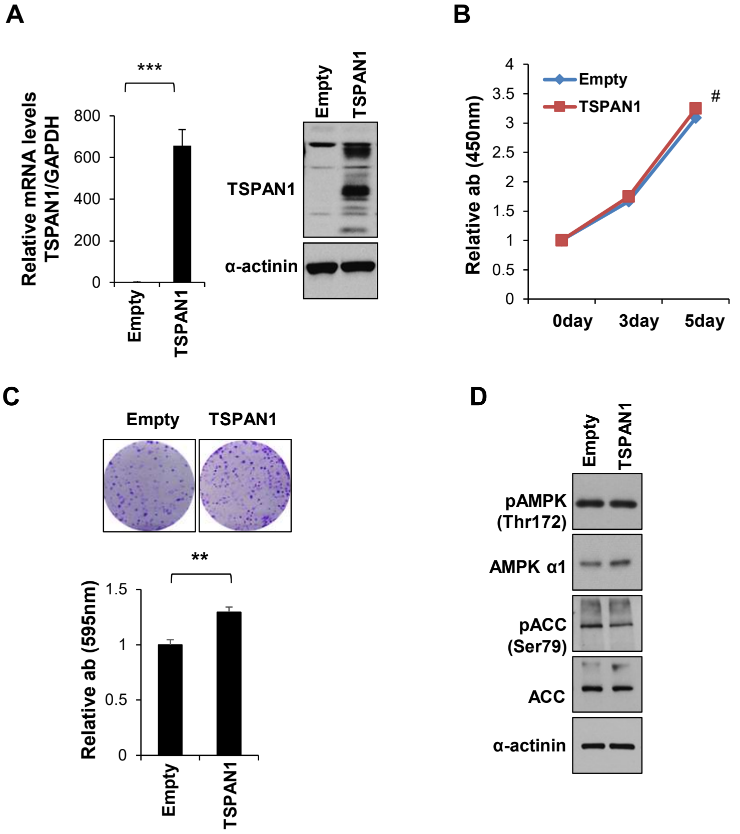

Fig. S5. TSPAN1 increases cell growth but not cell invasion in the TOV-21G stable cells.
A. TSPAN1 expression was detected by real-time PCR (left panel) and immunoblotting (right panel) in the TOV-21G stable cell line, with α-actinin as an internal loading control. Data are expressed as the mean ± standard error (S.E.); n = 3. Unpaired t-test was performed. ***p < 0.001. **B.** Cell proliferation was determined via WST-1 at various times in the TOV-21G stable cell line. Error bars represent mean ± S.E.; n = 3. **C.** Colony forming assay was performed in TOV-21G stable cells. Upper panel shows representative images; lower panel shows the relative absorbance at 595 nm. Error bars represent mean ± S.E.; n = 3. Unpaired t-test was performed. **p < 0.01. **D.** Protein expressions were detected using immunoblotting in the TOV-21G stable cell line with α-actinin as an internal loading control.
