# Supplementary material for: Tetraspanin 1 promotes endometriosis leading to ovarian clear cell carcinoma
Source: Mol Oncol. 2021 Jan 7;15(4):987–1004. doi: 10.1002/1878-0261.12884 (PMC8024726; doi:10.1002/1878-0261.12884)
Supplement: Supplementary file 6 — Fig. S6. In GSE53012 analysis, TSPAN1 expression was increased in cycling and chronic hypoxic conditions in PC‐3 and SKOV3, but not in WM793B. [file MOL2-15-987-s002.pdf]

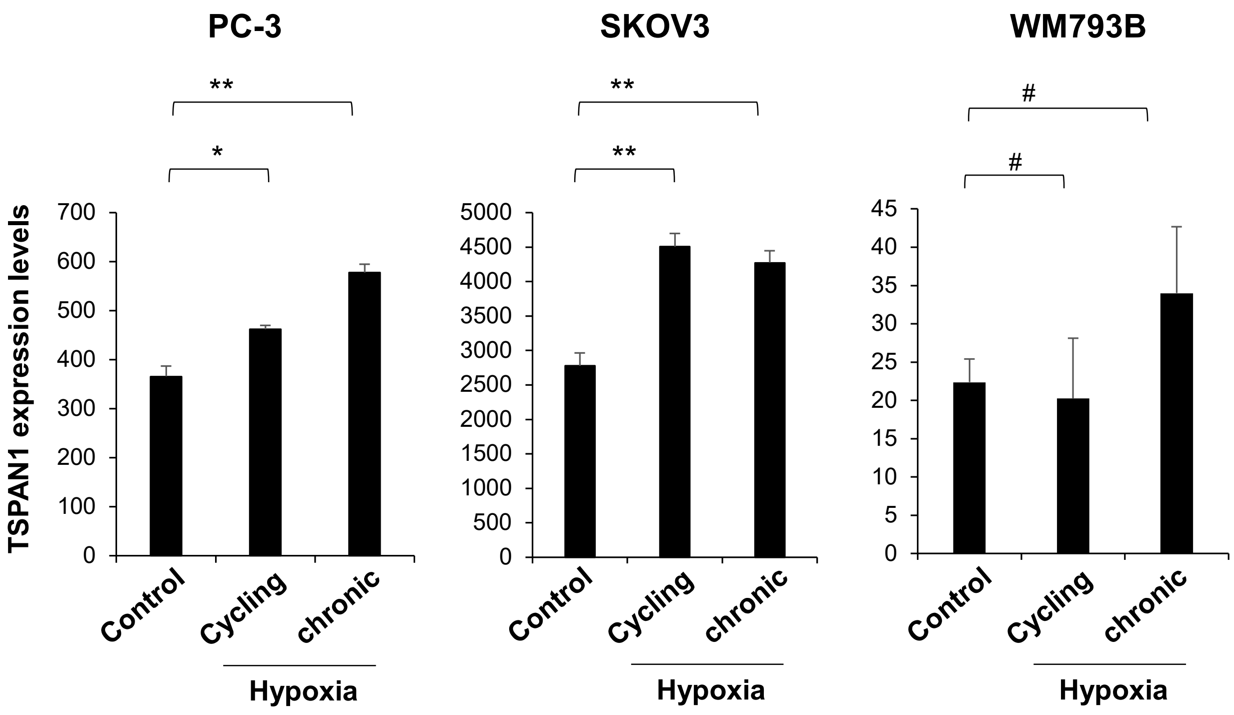

Fig. S6. In GSE53012 analysis, TSPAN1 expression was increased in cycling and chronic hypoxic conditions in PC-3 and SKOV3, but not in WM793B. Error bars represent mean ± standard error (S.E.). Unpaired t-test was performed. *$p < 0.05$, **$p < 0.01$.
