# Supplementary material for: Tetraspanin 1 promotes endometriosis leading to ovarian clear cell carcinoma
Source: Mol Oncol. 2021 Jan 7;15(4):987–1004. doi: 10.1002/1878-0261.12884 (PMC8024726; doi:10.1002/1878-0261.12884)
Supplement: Supplementary file 7 — Fig. S7. AMPK activity is high in OCCC and is increased by ARID1A knockdown. [file MOL2-15-987-s008.pdf]

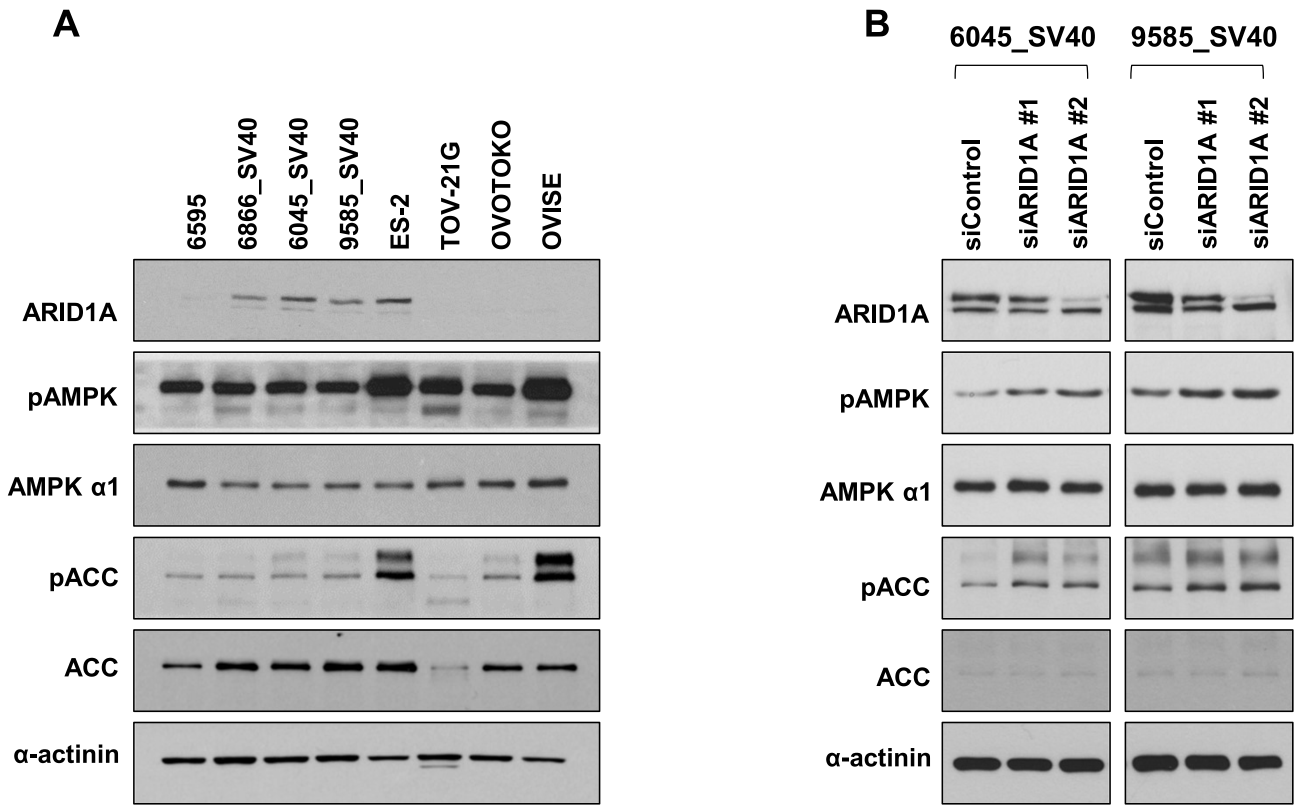

Fig. S7. AMPK activity is high in OCCC and is increased by ARID1A knockdown.

A. Protein levels of ARID1A, pAMPK (Thr172), AMPK alpha1, pACC (Ser79), and ACC were assessed via immunoblotting, with α-actinin as an internal loading control. **B.** Protein expressions were detected by immunoblotting after transient transfection of siControl or siARID1A in the 6045_SV40 and 9585_SV40 stable cell lines, with α-actinin as an internal loading control.
