# Supplementary material for: Tetraspanin 1 promotes endometriosis leading to ovarian clear cell carcinoma
Source: Mol Oncol. 2021 Jan 7;15(4):987–1004. doi: 10.1002/1878-0261.12884 (PMC8024726; doi:10.1002/1878-0261.12884)
Supplement: Supplementary file 8 — Table S1. Tabular data shows the fold change of 14 genes in 5 different two‐group comparisons. Table S2. STR profiling. Table S3. TSPAN1 score of immunohistochemical staining. [file MOL2-15-987-s006.docx]

**Table S1. Tabular data shows the fold change of 14 genes in 5 different two-group comparisons.**

| **Gene Symbol** | **DESeq2** | | | | |  | **edgeR** | | | | |
| --- | --- | --- | --- | --- | --- | --- | --- | --- | --- | --- | --- |
|  | **Endometriosis**  **vs AtyEm** | **Endometriosis vs AdjEm** | **Endometriosis vs OCCC** | **AtyEm vs OCCC** | **AdjEm vs OCCC** |  | **Endometriosis**  **vs AtyEm** | **Endometriosis vs AdjEm** | **Endometriosis vs OCCC** | **AtyEm vs OCCC** | **AdjEm vs OCCC** |
|  | Fold change (*P*) | Fold change (*P*) | Fold change (*P*) | Fold change (*P*) | Fold change (*P*) |  | Fold change (*P*) | Fold change (*P*) | Fold change (*P*) | Fold change (*P*) | Fold change (*P*) |
| **TSPAN1** | 2.4 | 4.646 | 80.747 | 33.532 | 17.381 |  | 3.412 | 7.065 | 101.098 | 2.608 | 4.494 |
|  | (0.048) | (0.005) | (0.001) | (1.E-05) | (0.006) |  | (0.045) | (0.005) | (0.001) | (4.E-05) | (0.008) |
| **EPCAM** | 2.352 | 4.431 | 54.089 | 22.994 | 12.206 |  | 2.534 | 4.411 | 47.906 | 18.906 | 10.859 |
|  | (0.048) | (0.001) | (0.001) | (1.E-05) | (0.006) |  | (0.044) | (0.001) | (0.001) | (1.E-05) | (0.006) |
| **TMEM184A** | 3.152 | 4.520 | 37.653 | 11.945 | 8.331 |  | 9.457 | 12.896 | 116.595 | 12.329 | 9.041 |
|  | (0.015) | (4.E-04) | (0.004) | (2.E-04) | (0.021) |  | (0.02) | (0.001) | (0.003) | (1.E-04) | (0.016) |
| **PKP3** | 2.061 | 3.133 | 30.603 | 14.849 | 9.768 |  | 3.735 | 6.723 | 66.888 | 17.910 | 9.949 |
|  | (0.029) | (0.004) | (9.E-05) | (1.E-07) | (0.001) |  | (0.034) | (0.005) | (1.E-05) | (4.E-08) | (4.E-04) |
| **ERBB3** | 2.020 | 2.375 | 26.721 | 13.228 | 11.250 |  | 3.874 | 5.502 | 76.145 | 19.655 | 13.838 |
|  | (0.011) | (0.014) | (3.E-04) | (1.E-06) | (0.002) |  | (0.019) | (0.018) | (2.E-04) | (1.E-06) | (0.002) |
| **MUC20** | 2.577 | 2.215 | 22.674 | 8.799 | 10.236 |  | 3.683 | 3.011 | 29.987 | 8.143 | 9.958 |
|  | (0.021) | (0.016) | (0.004) | (2.E-06) | (0.013) |  | (0.022) | (0.015) | (0.003) | (2.E-04) | (0.013) |
| **B4GALNT3** | 2.101 | 3.179 | 22.119 | 10.527 | 6.958 |  | 3.166 | 4.853 | 36.086 | 11.398 | 4.433 |
|  | (0.016) | (0.001) | (1.E-04) | (2.E-07) | (0.001) |  | (0.019) | (0.001) | (4.E-05) | (1.E-07) | (0.003) |
| **B3GNT3** | 2.044 | 2.119 | 18.426 | 9.015 | 8.695 |  | 4.695 | 5.452 | 63.170 | 13.456 | 11.587 |
|  | (0.023) | (0.014) | (0.001) | (1.E-05) | (0.004) |  | (0.041) | (0.012) | (0.001) | (9.E-06) | (0.004) |
| **EPS8L1** | 2.035 | 2.292 | 15.016 | 7.378 | 6.552 |  | 3.456 | 3.262 | 20.725 | 5.996 | 6.354 |
|  | (0.046) | (0.014) | (0.003) | (2.E-04) | (0.014) |  | (0.043) | (0.013) | (0.001) | (8.E-05) | (0.009) |
| **KRT19** | 2.181 | 2.521 | 13.117 | 6.014 | 5.204 |  | 2.179 | 2.705 | 11.992 | 5.503 | 4.433 |
|  | (0.022) | (0.021) | (4.E-04) | (4.E-04) | (0.004) |  | (0.019) | (0.019) | (2.E-04) | (3.E-06) | (0.003) |
| **BSPRY** | 2.051 | 2.306 | 11.844 | 5.774 | 5.136 |  | 4.150 | 4.758 | 33.224 | 8.007 | 6.983 |
|  | (0.007) | (0.002) | (0.001) | (2.E-05) | (0.007) |  | (0.01) | (0.002) | (0.001) | (3.E-05) | (0.024) |
| **SYTL1** | 2.442 | 2.543 | 11.776 | 4.821 | 4.631 |  | 3.586 | 3.594 | 17.433 | 4.861 | 4.851 |
|  | (0.007) | (0.004) | (4.E-04) | (1.E-05) | (0.004) |  | (0.006) | (0.003) | (2.E-04) | (1.E-05) | (0.004) |
| **SGK2** | 2.122 | 2.216 | 10.996 | 5.181 | 4.961 |  | 2.550 | 2.669 | 15.488 | 6.074 | 5.803 |
|  | (2.E-04) | (0.003) | (0.011) | (0.001) | (0.038) |  | (2.E-04) | (0.003) | (0.008) | (0.001) | (0.035) |
| **CDK2AP2** | 2.011 | 2.061 | 4.597 | 2.286 | 2.231 |  | 2.145 | 2.241 | 4.945 | 2.306 | 2.206 |
|  | (5.E-06) | (0.002) | (0.001) | (2.E-04) | (0.023) |  | (4.E-06) | (0.001) | (4.E-04) | (2.E-04) | (0.022) |

*P* values were performed by unpaired *t*-test.

**Table S2. STR profiling**

| **Locus** | **6595** | **6866_SV40** | **6045_SV40** | **9585_SV40** |
| --- | --- | --- | --- | --- |
| **D5S818** | 11, 13 | 10 | 12 | 10, 11 |
| **D13S317** | 9 | 10, 11 | 8, 11 | 8, 9 |
| **D7S820** | 11, 12 | 8 | 10, 11 | 10, 11 |
| **D16S539** | 11, 12 | 12 | 9, 12 | 9, 11 |
| **vWA** | 16, 18 | 17 | 16, 19 | 14, 17 |
| **TH01** | 7, 9 | 9 | 7, 9 | 8, 9.3 |
| **TPOX** | 8, 11 | 11 | 8, 11 | 8 |
| **CSF1PO** | 10 | 10 | 9, 10 | 12 |
| **AMEL** | X | X | X | X |
| **D3S1358** | 16 | 15, 17 | 15 | 15, 16 |
| **D21S11** | 30 | 30 31 | 29, 32 | 29, 31.2 |
| **D18S51** | 12, 17 | 16, 19 | 14, 24 | 12, 13 |
| **D8S1179** | 11, 14 | 10 | 13, 14 | 13, 15 |
| **FGA** | 21, 26 | 21, 22 | 21, 24 | 21, 22 |
| **D2S1338** | 23 | 19, 23 | 19, 24 | 17, 18 |
| **D19S433** | 14, 14.2 | 13, 14.2 | 13, 14 | 13, 15.2 |
| **Penta D** | 12, 14 | 9 | 9, 12 | 12 |
| **Penta E** | 12, 17 | 5, 20 | 15, 19 | 12, 19 |

**Table S3. TSPAN1 score of immunohistochemical staining**

|  |  |  | **Number of Patients** | **%** | **Mean** | **95% CI** | ***p* value** |
| --- | --- | --- | --- | --- | --- | --- | --- |
| **All study subjects** | | | 204 | 100 | 5.29 | (3.96-5.41) |  |
| **Diagnostic category** | | |  |  |  |  | **< 0.0001^a^** |
|  | Em | | 83 | 40.7 | 2.95 | (2.08-3.82) |  |
|  | AtyEm | | 13 | 6.4 | 6.00 | (3.66-8.34) |  |
|  | AdjEm | | 4 | 2.0 | 6.25 | (-0.42-12.92) |  |
|  | OCCC | | 51 | 25.0 | 7.07 | (5.85-8.29) |  |
|  | OEC | | 53 | 26.0 | 5.29 | (3.90-6.69) |  |
| **OCCC** | | |  |  |  |  |  |
|  | **FIGO stage** | |  |  |  |  | 0.0864^b^ |
|  |  | I | 25 | 49.0 | 5.89 | (4.04-7.73) |  |
|  |  | II/III/IV | 22 | 43.1 | 8.06 | (6.03-9.80) |  |
|  |  | Recurrent | 2 | 3.9 | 6.50 | (-63.38-76.38) |  |
|  |  | Unknown | 2 | 3.9 | 7.13 | (-16.7-30.95) |  |
|  | **Chemoresponse** | |  |  |  |  | 0.4491^b^ |
|  |  | Resistance | 7 | 13.7 | 5.12 | (1.13-9.10) |  |
|  |  | Sensitive | 24 | 47.1 | 6.57 | (4.70-8.43) |  |
|  |  | Unknown | 20 | 39.2 | 7.91 | (5.97-9.83) |  |
|  | **CA-125 (IU/L)** | |  |  |  |  | 0.8365^b^ |
|  |  | < 35 | 14 | 27.5 | 6.58 | (4.13-9.04) |  |
|  |  | ≥ 35 | 36 | 70.6 | 6.87 | (5.39-8.35) |  |
|  |  | Unknown | 1 | 2.0 | 12 | (0.00-0.00) |  |
| **OEC** | |  |  |  |  |  |  |
|  | **FIGO stage** | |  |  |  |  | **0.0362^b^** |
|  |  | I | 20 | 37.7 | 7.3 | (4.90-9.76) |  |
|  |  | II/III/IV | 28 | 52.8 | 4.3 | (2.46-6.04) |  |
|  |  | Recurrent | 1 | 1.9 | 0 | (0.00-0.00) |  |
|  |  | Unknown | 4 | 7.5 | 3.8 | (-5.29-12.79) |  |
|  | **Chemoresponse** | |  |  |  |  | 0.3164^b^ |
|  |  | Resistance | 2 | 3.8 | 7.5 | (-11.56-26.56) |  |
|  |  | Sensitive | 28 | 52.8 | 4.1 | (2.27-5.88) |  |
|  |  | Unknown | 23 | 43.4 | 6.6 | (4.23-8.95) |  |
|  | **CA-125 (IU/L)** | |  |  |  |  | 0.9381^b^ |
|  |  | < 35 | 7 | 13.2 | 5.1 | (-0.05-10.33) |  |
|  |  | ≥ 35 | 41 | 77.4 | 5.3 | (3.73-6.88) |  |
|  |  | Unknown | 5 | 9.4 | 5.4 | (-2.23-13.03) |  |

The values in bold are statistically significant. The unknown and recurrent were excluded at statistical analysis.

^a^ The value was analyzed by ANOVA test.

^b^ The values were analyzed by unpaired t test.

CI, confidence interval; FIGO, International Federation of Gynecology and Obstetrics; CA-125, carbohydrate antigen 125
